# Determination of anti-HCV and quantification of HCV-RNA and IP-10 from dried blood spots sent by regular mail

**DOI:** 10.1371/journal.pone.0201629

**Published:** 2018-07-31

**Authors:** Bastian Neesgaard, Morten Ruhwald, Henrik B. Krarup, Nina Weis

**Affiliations:** 1 Department of Infectious Diseases, Copenhagen University Hospital, Hvidovre, Denmark; 2 Center for Vaccine Research, Section of Human Immunology, Statens Serum Institut, Copenhagen, Denmark; 3 Section of Molecular Diagnostics, Clinical Biochemistry, Aalborg University Hospital, Aalborg, Denmark; 4 Department of Clinical Medicine, Faculty of Health and Medical Sciences, University of Copenhagen, Copenhagen, Denmark; Saint Louis University, UNITED STATES

## Abstract

**Background:**

With the introduction of direct acting antivirals, treatment of hepatitis C virus (HCV) in hard-to-reach populations is now feasible. Therefore, new cost-effective and reliable test methods are needed. Determination of HCV antibodies and HCV-RNA from dried blood spots samples could represent one such method. Here we examined whether anti-HCV could be detected—and HCV-RNA quantified—from dried blood spots, sent by regular mail. We also investigated, if IP-10 determined from dried blood spots correlated with fibrosis progression appraised by transient elastography.

**Method:**

Forty chronic HCV infected patients were consecutively enrolled. At baseline and after six months, dried blood spots were prepared from blood collected by venous puncture, dried for 4–6 hours, then stored in gas-impermeable plastic bags with a desiccator, before being sent by regular mail. At each visit, approximately six months apart, paired venous samples was obtained and analyzed for anti-HCV, HCV-RNA and IP-10.

**Results:**

Anti-HCV was found in 66/67 of the dried blood spots. Sixty-six paired samples were available for HCV-RNA analysis. A statistically significant correlation was found between log HCV-RNA concentrations in plasma, and log HCV-RNA obtained from (P < 0.0001, Pearson’s R 0.6788, R^2^ 0.4607). HCV-RNA, derived from DBS samples, was lower than the corresponding plasma concentration, reflected by a Bland-Altman bias of 3 with SD of bias ± 0.6472. We found no correlation between IP-10 and fibrosis progression.

**Conclusions:**

We identified anti-HCV in 66/67samples, and quantified IP-10 and HCV-RNA from dried blood spots, dried at room temperature and sent by regular mail. HCV-RNA concentrations from the dried blood spots were lower than corresponding plasma values; a probable result of heparin coated test tubes. We found no correlation between IP-10 and fibrosis progression. Overall, dried blood spots could be a cost-effective and easy-to-use alternative to standard tests for the diagnosis of HCV infections.

## Introduction

Globally, the prevalence of chronic hepatitis C (CHC), defined as detectable hepatitis C virus (HCV)-RNA in two consecutive measurements six months or more apart, is estimated to 71 million people [1], with CHC being a leading cause of chronic liver disease [2]. CHC can lead to liver fibrosis, which may in turn lead to the development of cirrhosis in approximately 5–20% of cases [2–5]. Once cirrhosis occurs, there is a marked increase in the risk of developing hepatocellular carcinoma [6].

With the introduction of Direct Acting Antivirals (DAAs), treatment regimens are now all-oral, with shorter duration, higher rates of sustained virological response and fewer adverse effects. Thus, treatment of populations that have previously been viewed as hard to reach is now feasible.

With these new treatment possibilities, new cost-effective, easy to perform and reliable methods are needed to increase the diagnostic rate of HCV infected people in these difficult-to-reach populations.

Dried blood spots (DBS) could represent such a method. Since their introduction in the early 1960s, by Dr. Robert Guthrie as a simple screening test for phenylketonuria in newborns [7], the use of DBS has since been extended to aid in the diagnosis of a wide variety of pathogens, including hepatitis A, B and HIV [8, 9]. Detection of HCV-RNA [10–21], anti- HCV [9, 20, 22–25] and determination of HCV genotype (GT) [15, 19, 21] from DBS have also been investigated. Although HCV-RNA can be detected at levels of 250 IU/mL in DBS [11], this is still higher than the 50 IU/mL detection limits for plasma samples. Despite findings of a strong correlation between the detection of HCV-RNA in plasma samples and DBS [21] it remains unclear if DBS can be used as a valid tool for quantification of the viral load. In addition, it is unclear how storage, temperature and handling of DBS affect the levels of HCV-RNA.

Recently, within our group, a method to accurately measure interferon γ-induced protein 10 kDa (IP-10) in DBS by ELISA was developed [26]. Also we have recently showed that GT 1 infected patients with cirrhosis have significantly higher levels of IP-10 than patients with no- or mild fibrosis [27, 28], fully in line with others’ results for HCV GT1 infected patients [29–33]. Together with the above-mentioned previous findings on HCV-RNA, the implication of DBS use could hold a promising future for diagnosing HCV infections and surveillance of disease activity. However, this is dependent on antibodies and HCV-RNA stability on DBS, in order to be successfully analyzed after being sent / shipped from the patient to the laboratory facilities.

The primary objectives of this study were to examine if anti-HCV antibodies could be detected and HCV-RNA and IP-10 quantified in DBS samples sent by regular mail, and how the HCV-RNA value from these samples related to the HCV-RNA value in paired plasma samples. As a secondary objective, IP-10 values in DBS were correlated to paired plasma samples, and IP-10 correlated to the degree of liver fibrosis, as determined by Transient Elastography.

## Method

### Ethics statement

This study was approved by The Regional Committee on Biomedical Research Ethics (H-3-2013-152). After oral and written information about the study all patients gave informed consent in writing.

### Patients

Patients’ ≥ 18 years of age were consecutively enrolled from the outpatient clinic, Department of Infectious Diseases, Copenhagen University Hospital, Hvidovre, Denmark. Individuals were considered for inclusion if they had CHC (detectable HCV-RNA in two consecutive measurements six months or more apart). Patients were excluded if they were co-infected with HIV or HBV, had alcohol dependency- (regular intake of ≥ 75 g/day), ongoing intravenous drug use, had been treated with immunosuppressant drugs or had received treatment for CHC within the last six months.

At baseline, we registered patients’ gender, age, ethnicity, anti-HCV, HCV-RNA, HCV-genotype, IP-10, ALT levels and Transient Elastography (TE) score.

### Sample collection and preparation

#### Preparation of DBS—And plasma samples

On the day of enrollment, DBS samples for IP 10 analyses were prepared, as described in detail elsewhere [26]. In brief, the skin on a random fingertip was pierced by a small finger prick, after the skin had been disinfected by a standard ethanol swipe. Blood was then added to a filter paper (903 Protein Saver™ cards, Whatman) in duplicate. After 4–6 hours’ drying on the lab bench DBS samples were stored in a gas-impermeable plastic bag with a desiccator and placed in another gas-impermeable plastic bag. The samples were then placed in a standard envelope for transferring biological material and sent by regular mail to the Department of Infectious Diseases, Copenhagen University Hospital, Hvidovre, Denmark. On arrival, the filter paper containing the IP-10 samples were frozen at -20 OC until time of analysis. Denmark is located in the temperate climate zone. During the study period, the average temperature therefore was between 0.5–16°C. The average transit time in mail was 4 days.

At the same day as DBS preparation, a venous blood sample was drawn, using a 4 mL Li-Hep coated tube containing a separation gel. Forty μl blood (per spot) were transferred in duplicates to another piece of filter paper, intended for HCV-RNA quantification and antibody determination. Hereafter, the plasma was separated from the whole blood by centrifuge and 0.5 mL were transferred to a cryotube, and frozen at -20 OC until time of analysis for plasma IP-10 levels. The filter paper containing the microbiological samples was dried, packed and sent, in the same manner as the IP-10 samples, however, on arrival these were frozen at -80 OC. At the patient’s next routine visit, six months later, this procedure was repeated.

#### Transient elastography

For liver stiffness measurements we used Fibroscan® (Echosens, Paris, France). Transient Elastography (TE) is a novel, noninvasive and rapid bedside method to assess liver fibrosis. During the first round of tests, TE was performed on the same day as DBS and blood samples preparation for 38 of the 40 patients—and within a week for the two remaining patients. All TEs were performed on the day of DBS and blood sample preparation during the second round of samples.

Patients were diagnosed as having no or mild fibrosis when TE showed a median liver stiffness ≤ 7.7 kPa, moderate fibrosis with stiffness 7.7 kPa-13.3 kPa, and as having cirrhosis when the values were ≥ 13.3 kPa, in accordance with a previous large meta-analysis examining correlations between fibrosis determined by liver biopsy and TE [34]. Liver stiffness measurements were performed by physicians or nurses certified for the use of TE and with experience from more than 50 examinations.

### HCV-RNA determination and anti-HCV

HCV-RNA from DBS was extracted with either lysis buffer, protease K or plain, purified H2O. Plain water turned out to give the best yield with our RNA extraction procedure. One DBS was dissolved in 1mL H2O overnight. The samples were spun down at 10.800 G for 10 minutes.

We tested 500 and 100 μL of the supernatant, and 100 μL gave the best yield, whereas 500 μL resulted in some inhibition. Total RNA was isolated from 100 μL supernatant using NucliSENS easyMAG Magnetic Silica (bioMérieux, Marcy l’Etoile, France). Detection and quantification of viral nucleotide sequences were performed by In house real-time PCR using molecular beacons on a Mx3005P Real-Time PCR System (Stratagene, La Jolla, USA). We also tested our standard routine method Abbott RealTime HCV, but were not able to get meaningful results. Antibodies against HCV (anti-HCV) were tested using our routine chemiluminescence assay (anti-HCV, Architect; Abbott Laboratories, Abbott Park, IL, USA) according to the manufacturer’s recommendation only using supernatant instead of plasma.

### IP-10 measurements

IP-10 levels in plasma and DBS samples were determined using ELISA, developed and optimized for the monitoring of IP-10 in plasma and filter paper samples [26]. In brief, when used for DBS’s, 2 discs of 5.5 mm are punched from the center of the DBS using a standard office paper puncher (Impega, UK) and incubated with 100 μL assay buffer. Plasma samples were diluted 1:4 in assay buffer. Plasma and DBS discs were incubated for 2 hours at room temperature (23 OC) and washed x 3, where after HRP-substrate (TMB One, Trichem) was added and plates were revealed for 30 minutes before the color reaction was stopped with 100 μL H2SO4 and absorbance was read Concentrations were calculated using a standard curve with a linear range from 2.5–600 pg/ml (Peprotec, USA). Plasma concentrations were corrected for the dilution factor (multiplied x 5).

### Statistics

We used Mann-Whitney or Kruskal-Wallis when appropriate to uncover association between baseline parameters and IP-10. Linear regression was performed to show correlations between plasma and DBS values and indicating cohesive r–and r^2^ values. Bland-Altman plots were used to compare analytical methods. P values < 0.05 were considered statistically significant.

## Results

In all we included 40 patients at baseline. The majorities of the patients were White, and infected with GT 1. Overall baseline demographics and clinical variables for study patients can be found in Table 1.

**Table 1:** Baseline characteristics for study patients.

Number of patients (n)	40
Gender (M/W) (n)	19/21
Age (years) (median, range)	55 (32–67)
Ethnicity (White /Hispanic /Asian)	35/2/3
TE-score (<7.7 kPa / >7.7 kPa—<13.3 kPa / ≥ 13.3 kPa) (n)	26/7/7
ALT (IU/L)* (median, 25% - 75% percentile)	53 (34–95,5)
Genotype (1/2/3) (n)	25/5/10
HCV-RNA (IU/ml) (Mean, CI 95%)	687.000 (443.000–1.129.000)
Plasma IP-10 (pg/mL) (mean, CI 95%)	279 (92–366)

*ALT levels could be obtained for 37/40 patients

### HCV-RNA

**Fig 1:**
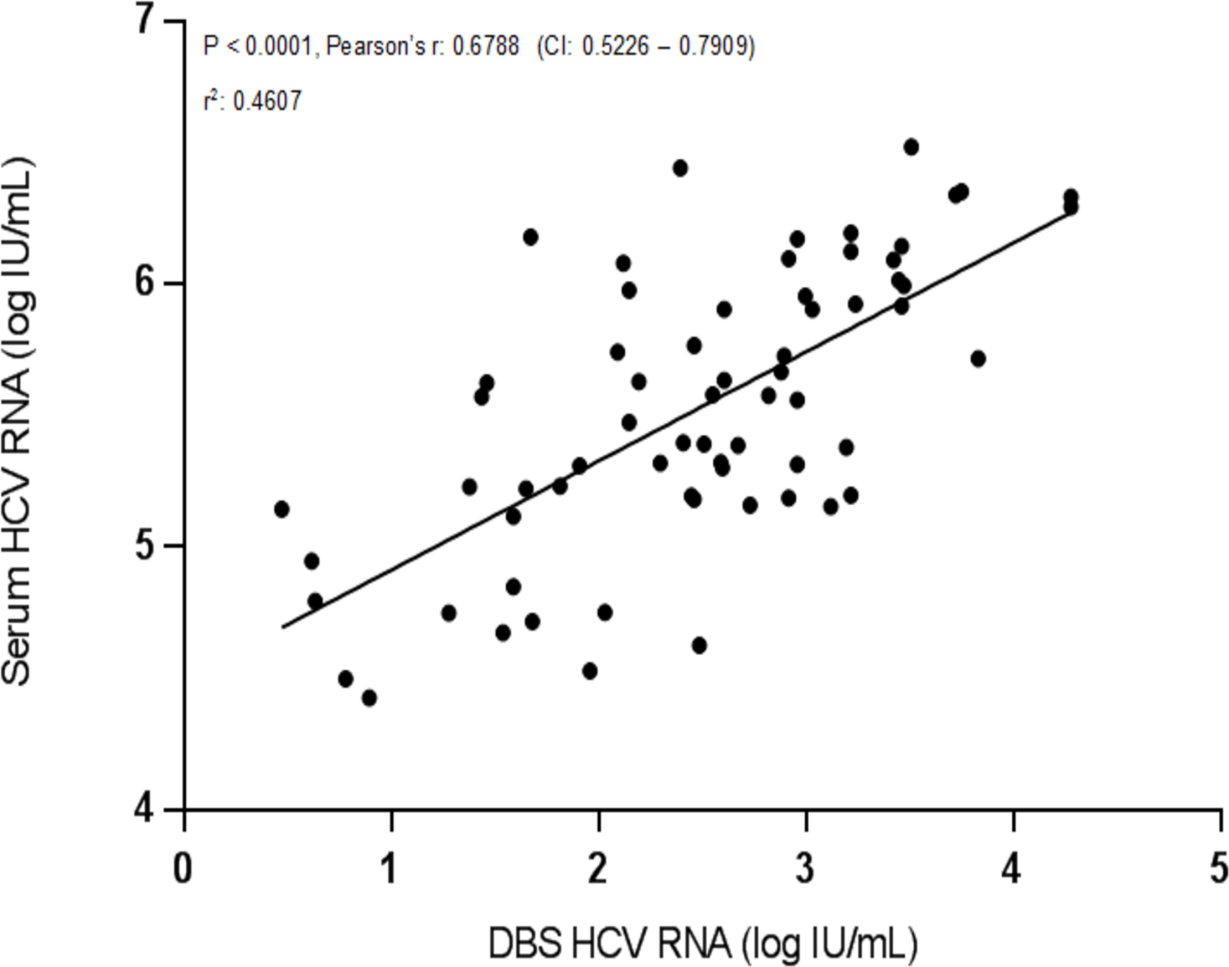
Linear regression showing correlation between HCV-RNA Concentrations in plasma and DBS samples.

Eleven patients were lost to follow up, initially leaving 69 parried samples from 40 patients available. One DBS sample from baseline was incorrectly prepared and discarded. One DBS sample and one plasma sample from two individual patients were false negative, reflected by finding no HCV-RNA in patients known to be HCV-RNA positive. Exclusion of these three samples (and the corresponding plasma or DBS sample) left 66 paired samples for analysis. Fig 1 shows a statistically significant correlation between log HCV-RNA concentration found in plasma and log HCV-RNA deviated from DBS (P < 0.0001, R 0.6788, R^2^ 0.4607).

**Fig 2:**
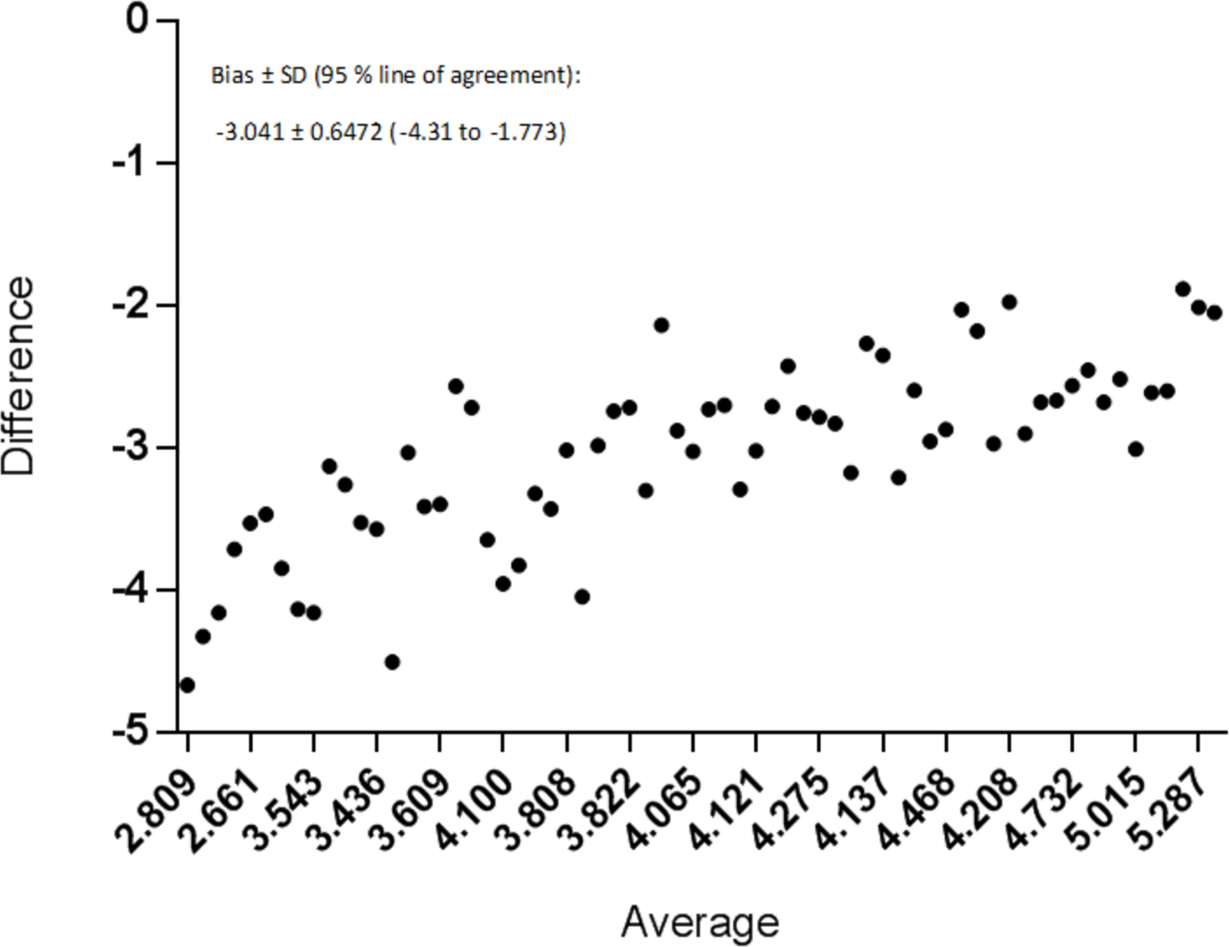
Bland-Altman plot showing difference and average of samples derived from Log plasma HCV-RNA concentrations and Log DBS HCV-RNA concentrations.

This association remained when baseline samples and 6 months samples were analyzed separately (P < 0.0001, R 0.7526, R^2^ 0.5664 and P < 0.0001, R 0.5754, R^2^ 0.4562 respectively). The HCV-RNA concentrations measured in DBS samples were systematically lower than the corresponding plasma levels, as illustrated by the Bland-Altman plot showing a bias of -3.041 log HCV-RNA with SD of bias ± 0.6472 (Fig 2).

The plot also shows the difference being highest at the lower values.

### Anti-HCV

Samples from 67 patients were available for anti-HCV determination. As all patients included in the study were HCV-RNA positive, we expected all 67 samples to be anti-HCV positive. We found 66 of the samples to be anti-HCV positive, and one sample to be (false) negative, translating to a sensitivity of 98.5%.

### IP-10 and fibrosis stage

All 40 patients underwent a Fibroscan at baseline with a median TE-score of 6.1 kPa (25% percentile– 75% percentile, 4.9–10.2). Twentynine patients were available for follow up 6 months later with a median score of 5.6 kPa (25% percentile– 75% percentile, 4.7–8.6). There was no difference in the TE scores between baseline and 6 months follow up (P = 0.4736). Out of the original 40 patients included, we were able to acquire subsequent TE-scores from 17 patients performed 12–24 months after baseline with a median score of 7.6 kPa (25% percentile– 75% percentile, 6.1–8.9). We found no significant difference in baseline IP-10 levels when comparing patients with TE-scores above or below 7.7 kPa. (P = 0.267). Table 2 shows that patients who showed any increase in TE score, between baseline and the six months follow up, did not have higher baseline plasma IP-10 than patients who showed any decrease in TE score values during the six months period (P = 0.266). Splitting patients up into groups, based on the baseline plasma IP-10 levels (<100 pg/ml, 150–300 pg/mL and > 300 pg/mL), did not yield any significant differences in these patients’ TE scores at baseline (P = 0.231).

**Table 2:** Median plasma IP-10 concentrations at baseline in relation to genotype, ALT levels, TE-score levels- and dynamics and plasma HCV-RNA concentration.

		Samples (n)	Median [IP-10] (pg/mL)	25% - 75% percentile (pg/mL)	P-Value
Genotype:	GT 1	25	263	154–389	0.031
	GT Non-1	15	141	116–287	
ALT *	ALT > 40 IU /mL	26	170	136.3–343	0.507
	ALT < 40 IU /mL	11	250	125–351	
TE score at baseline	< 7.7 kPa	27	170	125–351	0.267
	> 7.7 Kpa	13	278	160–341	
Changes in TE score^¤^	Increase in TE score	14	304	153–394	0.266
	Decrease in TE score	15	188	140–338	
HCV-RNA ^#^	< 1 x 10 ^6^ IU/mL	27	170	125–310	0.421
	> 1 x 10 ^6^ IU/mL	12	288	109–379	

*Three patients did not have available ALT data. ^¤^ Increase–or decrease in TE-Score value between measurements at baseline and at follow up 6 months after. Twenty-nine patients completed follow up.

^#^ One plasma HCV-RNA sample was incorrectly analyzed and excluded from analysis.

We examined if IP-10 concentrations at baseline correlated with HCV GT, ALT, TE-scores and plasma HCV-RNA concentration (Table 2).

We found a significantly higher plasma IP-10 concentration in HCV GT 1 infected individuals, as opposed to genotype-non-1 infected, but no other correlations.

**Fig 3:**
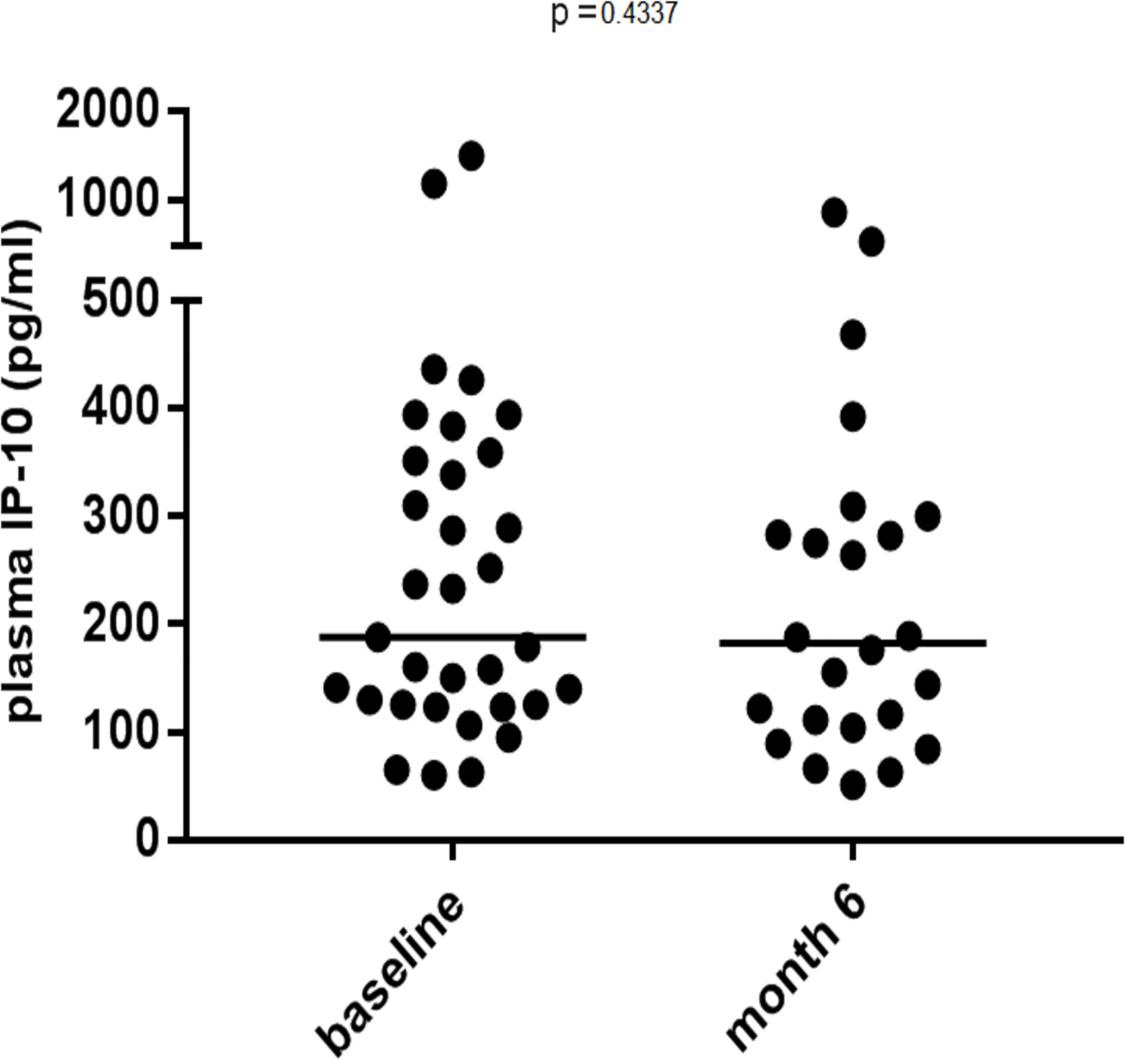
Showing no significant difference in plasma IP-10 levels in samples derived from baseline and at 6 months follow-up.

Due to a strong suspicion of a technical problem in the analysis of the first seven DBS samples originating from baseline, (wrong buffer added), we excluded these samples. To allow paired analysis the corresponding plasma samples were also excluded from the analysis. Two DBS IP-10 samples failed at preparation at follow up, leaving 60 paired samples for further analysis (the original 69 paired samples minus the 9 paired samples). When comparing IP-10 concentrations on DBS and paired plasma samples, we found a statistical significant correlation (P < 0.0001, Persons r = 0.526, r^2^ = 0.2677). The corresponding Bland-Altman plot showed a bias of 219.9 pg/mL (SD ± 238.9) and some heteroscedasticity in the low concentration range as expected from lower volume available in the DBS samples. Fig 3 shows that we found no statistical significant difference in plasma IP-10 levels between baseline and 6 months follow-up.

## Discussion

In this study we explored the potential of using DBS samples, sent by regular mail to determine Anti-HCV antibodies, HCV-RNA and IP-10. We found statistically significant correlations between log HCV-RNA and IP-10 concentration found in plasma and DBS, as well as successfully detected anti-HCV in 66/67 DBS samples.

Our findings on HCV-RNA are in line with previous studies [21, 35]. The difference in HCV-RNA levels between plasma and DBS samples in this study were however lower than earlier findings (1.5 log IU/mL and 2.27 log IU/mL). The difference could be due to HCV-RNA lost from the samples as they were transported by regular mail. Also, some of the difference could be due to a lower number of patients in our study or variations in the methods used to analyze the samples. Another reason could be the use of 903 Protein Saver™ cards, where FTA cards would presumably have been more ideal for RNA analyses. However, as we used a single heparin coated tube—known inhibitor of PCR, for extracting blood for both creating IP-10 plasma samples and later PCR, it is very likely that this is the main reason for the lower concentrations found.

Detection of HCV-RNA without quantification has also been shown in a number of previous studies [10–13, 15, 16, 19, 20], with sensitivity and specificity up to 90% and 100% with corresponding PPV and NPV of 100% and 94% [13] The conjoined findings of correlation between plasma and DBS samples, with- or without HCV-RNA quantification, indicate the DBS samples can reliably be used to detect HCV-RNA. This report further adds that samples can be mailed at ambient temperatures by regular postal services. Only one of the DBS samples, from a known HCV-infected patient, failed to show any HCV-RNA when tested. Regarding anti-HCV we here confirm previous findings. [13–17, 20–22] Out of the 67 samples available from HCV-RNA positive individuals, 66 were antigen positive. Corresponding to a sensitivity of 98,5%.

In the era of pangenotypical DAA treatment, the need for quantifying HCV-RNA is hastily diminishing, as HCV-RNA levels no longer have any role in treatment algorithms. From a clinical point of view the information needed is thus: Is the patient HCV-RNA positive following a positive anti-HCV test? Detection limits previously described for HCV-RNA in DBS range from 150 [11]–to 2500 IU/mL [12], which is below the value in most patients with CHC [36]. Thus, DBS testing represents an easy-to-use, stable test method to determine anti-HCV, and HCV-RNA status in hard-to reach-settings, as well as in hard-to-reach populations. Therefore, it could be a valuable tool for reaching the WHO goals for diagnosing 90% of HCV infected individuals by 2030.

In this study we found no significant difference in baseline IP-10 levels and TE values, though the relation between fibrosis and high IP-10 levels had been well documented [28, 31, 33, 37]. Regarding IP-10’s ability to predict fibrosis progression or decrease, we here only examined 39 patients in a course of six months. And only a small number of test participants had TE score values available 12–24 months after baseline. In the timespan we followed our patient group we did not find any significant changes in fibrosis levels. However, it would be interesting to follow up on fibrosis levels in 5–10 years, where significant changes in fibrosis could have occurred. Only a small proportion of patients in our examination were cirrhotic at baseline. Likewise, relatively few had advanced fibrosis. This, along with the limited number of patients included, is a reasonable explanation why we did not find any correlation, and previous findings [28, 31, 33, 37] should therefore not be questioned on the basis of this study. In any case, we here found a statistically significant association between IP-10 derived from plasma, and IP-10 derived from DBS. However, the strength of the correlation was only moderate, as reflected by the Pearson’s r of 0.526. Which does not represent satisfactory strength for clinical use. The results should though be interpreted in the light of this being a small study with only 40 patients included. It has previously been shown that IP-10 can be reliably measured in DBS [27, 28]. Here we did not include control samples which was not sent by mail. Therefore, we could not evaluate what effect samples being sent had on IP-10 levels. Which might have weakened the association we did find. This element should be added to future studies.

A strength of this study is that we consecutively enrolled patients from our outpatient clinic representing a standard cross section of Danish CHC patients, and this report is the first Danish study to successfully quantify HCV-RNA from DBS. In addition, this study is proof-of-concept that it is possible to do so from DBS samples sent by regular postal service.

The study has some limitations that need to be addressed. First, the number of patients included was relatively low. Secondly, we did not include any healthy controls making us unable to calculate NPP and PPV values.

Another limitation is that we used blood from venous tubes to create the DBS which could possibly yield some differences compared to DBS created from capillary blood, and that heparinization of the blood probably was the main reason for the lower concentrations of HCV–RNA found in blood compared to DBS samples. For the determination of fibrosis level, we depended solely on TE, with a possible under- or over-estimation of intermittent levels of fibrosis.

## Conclusions

We successfully quantified HCV-RNA from DBS samples, dried at room temperature and sent by regular mail. Concentrations of HCV-RNA from DBS were statistically significantly correlated with plasma concentrations HCV-RNA. However, HCV-RNA concentrations obtained from DBS were lower than the corresponding plasma values; a probable result of using heparin coated test tubes. We found 66/67 (98.5%) of HCV-RNA positive patients to be anti-HCV positive in blood derived from filter paper, with only one false negative sample. We found that IP-10 determined from DBS correlated statistically significantly with paired plasma samples. We did however not find any correlation between IP-10 concentrations and liver fibrosis.

Over all, DBS could be a cost-effective easy-to-use and reliable alternative to standard tests for the diagnosis of HCV infections.

## Supporting information

S1 Table Overview of data at baseline, 1^st^ and 2^nd^ round of testing. (XLS)
